# Do Authoritarian Governments Respond to Public Opinion on the Environment? Evidence from China

**DOI:** 10.3390/ijerph15020266

**Published:** 2018-02-04

**Authors:** Xiao Tang, Weiwei Chen, Tian Wu

**Affiliations:** 1 School of Public Policy and Management, Tsinghua University, Beijing 100084, China; tangxiao@mail.tsinghua.edu.cn (X.T.); 15201522774@163.com (W.C.); 2 Institute for Contemporary China Studies, Tsinghua University, Beijing 100084, China; 3 School of Economics and Management, University of Chinese Academy of Sciences, Beijing 100190, China; 4 Key Laboratory of Big Data Mining and Knowledge Management, Chinese Academy of Sciences, Beijing 100190, China; 5 Academy of Mathematics and Systems Sciences, Chinese Academy of Sciences, Beijing 100190, China

**Keywords:** environmental governance, public opinion, governmental responsiveness, China

## Abstract

Given its serious impacts on the public’s health, air pollution in China is a matter of strong public concern, particularly in reference to malodorous waste gas. Petition letters related to atmospheric pollution accounted for about 40% of the total petition cases. However, scholarly views differ on whether the Chinese government responds to public opinion on the environment and seeks to improve its environmental governance behavior. For this study, data from national surveys on the public’s environmental satisfaction administered during the period 2011–2015 were analyzed to determine whether the public’s dissatisfaction with the state of the environment in a given year resulted in increased investments by provincial governments in pollution governance during the following year. The study’s findings revealed that governmental behavior in response to public opinion on the environment was selective within the field of environmental governance, with provincial governments being inclined to invest more in waste gas pollution control than in water pollution control. Furthermore, results from this study show that the Chinese government tends to put more efforts into the environmental field where it could more easily achieve short-term benefits.

## 1. Introduction

An extensive haze of air pollution caused by China’s unsustainable energy structure has raised widespread concern given its serious impacts on public health, while the problem of CO_2_ emissions has captured the attention of several scholars [1,2]. Although consistent attempts have been made to develop new energy resources in China and to propel the transformation of China’s energy structure, coal still accounted for 62% of China’s total energy consumption in 2016 [3]. Various pollutants produced by burning coal such as nitrates, sulfates, sodium chloride, and mineral dust directly contribute to the severe haze pollution that has deeply impacted the public’s health. Based on pollution data obtained from 90 cities north and south of the Huaihe River over recent decades, Chen et al. (2013) found that exposure to polluted air over a long duration, entailing an increase of 100 μg/m^3^ in total suspended particulates, caused an increase in the death rate by 14% and a reduction in the average life expectancy by 3 years [4]. Furthermore, heart and lung diseases are almost always the underlying causes of the increased death rate. The haze-induced hazards in China have consequently aroused public attention. The China Environment Yearbook (CEY) showed that around 40% of petition letters focused on atmospheric pollution. This figure was more than 10% higher than that for the second key public concern, namely, water pollution. Starting from 5 June 2009 when the national environmental “12,369” hotline was launched, the percentage of reports and complaint calls relating to atmospheric pollution has been steadily rising from an initial level of 50% to the current level of around 70%.

However, within academic circles, there are differing viewpoints on whether the government responds to public opinion on the environment. Mol and Carter (2006) characterized China’s mode of environmental governance as a form of environmental authoritarianism. They argue that the Chinese public is not allowed to participate in the environmental policy development process and that there is no mechanism for channeling public involvement within such a process [5]. Consequently, the public cannot influence the development and implementation of environmental policies [6,7]. However, some scholars have pointed out that the Chinese government has established various mechanisms such as environment petitions and hearings on major environmental issues as well as to evaluate environmental impacts. These hearings have enabled the compilation of public opinion on environmental issues as a reference for environmental decision making [8,9]. Based on this compiled data, it has been argued that public opinion has affected governmental action relating to environmental governance [10].

This study investigated how environmental governance is influenced by public opinion through an analysis of the relationship between the level of dissatisfaction of the Chinese public relating to the environment and quantities of funds invested by provincial governments in the environment. The data for the study was obtained from several rounds of the public environmental satisfaction survey administered in China during the period 2011–2015. The survey results showed that public dissatisfaction has significantly influenced quantities of environment-related investments by provincial governments in China. Specifically, public dissatisfaction has had a significant impact on the investments of Chinese provincial governments in the area of atmospheric pollution governance but has had an insignificant impact on investments in the area of water pollution governance. These findings reveal that whereas the Chinese government does respond to public opinion on the environment, its behaviors are selective.

This study contributes to several strands of the literature. First, it augments the literature on environmental authoritarianism in China. Environmental authoritarianism is a mode of environmental governance generated in a context that entails a conflict between values such as freedom and democracy and sustainable development [11,12]. China’s environmental policy implementation has attracted much academic attention from scholars. “Environmental authoritarianism” argues that under the top–down target setting system, governments may effectively enhance environmental policy outcome without pressures imposed by environmental interest groups in democratic institutions [13,14]. It contends that the free-rider phenomenon might be avoided without the interference of interest groups [15]. As shown in various environmental governance practices, China has been considered an example of the environmental authoritarian model [5]. In such a system, an authoritarian leader imposes a number of measures aimed at limiting freedom and allocating resources to prevent public involvement in major disasters caused by competition over resources [16]. There are two ways of implementing environmental authoritarianism. The first entails limiting personal freedom, preventing behaviors adverse to sustainable development and forcing people to follow the sustainable development policy. The second entails a lack of public participation, with the overall policy development process controlled by just a few members of the political elite [17]. China’s system of environmental governance can be considered to be an apt example of environment authoritarianism for the following reasons. First, its national environmental policy development process exhibits features that are evidently monocentric. The five-year plans of central and local governments are the main documents guiding environmental governance in the country, with the overall planning of the central government being essential. Accordingly, decision making relating to environmental policy primarily occurs within the central government, and the public lacks any means or channel for participating in the policy development process [18]. Second, China’s environmental civil society is weak. Environmental Non-Governmental Organizations (NGOs) are marginalized within the political sphere, mostly playing an auxiliary role in facilitating the implementation of environmental policies and consequently having a very limited impact on the decision-making process [19]. Last, a set of environmental monitoring systems suited to the Chinese context, including command controls, marketization tools, public participation, and voluntary action has gradually evolved over the course of the development of China’s environmental governance process. Nevertheless, China’s environmental governance still relies on mandatory administrative regulatory means that are questionable because of the lack of public participation and protection of property rights that they afford [20]. This study offers an alternative perspective that incorporates an examination of the role of the public in China’s environmental management process. In a context of environmental authoritarianism, it is widely accepted that the influence of the public on environmental governance is very limited. However, this study further explores the effects of public satisfaction on environmental governance.

This study directly investigated public participation and the responsiveness of the Chinese government within the field of environment governance. Some scholars like Gilley (2012) claimed that the Chinese government limits the means for citizens to express their opinions and is not therefore responsive to them [7]. He points out that during the process of climate change policy formulation, there was no channel provided for the public’s participation either during the process of constructing policy frameworks or during their implementation. Because members of the public are completely cut off from environmental information channels, they cannot express their environmental opinions. However, the uninformed public generally recognizes the effects of country on climate change, further highlighting this feature of authoritarianism. Xie (2009) pointed out that a channel does exist for public participation but that this channel is mainly aimed at promoting policy implementation, with the public’s participation limited to cooperation with the government. Thus, members of the public find it difficult to participate in policy development. In other words, the view of these scholars is that the government only responds to public opinion on the environment that is in its favor and supports its objectives [6]. The results of a survey conducted by Sullivan and Lei (2009) revealed that within the Chinese environmental network, civil society members accounted for just 39% of the total membership, with governmental and social linking network patterns respectively accounting for 34% and 26% of the total membership [21].

However, other scholars have argued that public opinion has impacted the environmental governance behaviors of the Chinese government. Data extracted from the CEY showed that the total number of environmental petition cases increased nearly fivefold during the period 2000–2014 and that the government’s complaint handling and resolution rates remained above 95% during this period. Thus, environmental petitions have had an evident impact on the environmental governance behaviors of local governments [10]. Providing opinions on environment-related issues also constitutes an important channel for the public to influence the environmental policy development process. For example, approval of the revised Environment Protection Law was postponed because of intensive societal debate [22]. Wang and Wheeler (2000, 2005) were among the first scholars to conduct empirical research on the government’s responses to public opinion within the field of environmental governance. They analyzed the relationship between the number of environment-related petition letters and the amount of sewage charges. They found that higher sewage charges were imposed in areas where there were more complaints regarding environmental quality [23,24]. This was mainly associated with the imposition of charge relating to waste gas emission but not with the imposition of a waste water charge. The waste gas emission is mainly referred to as the air pollution in this study. This study expands on the work of Wang and Wheeler, adopting subjective indicators that represent public opinions as the research objects. By doing so, it provides an alternative analytical perspective that can deepen understanding regarding the responsiveness of the Chinese government to public opinion on environmental issues. However, this study mainly deals with the environment issues.

The two respective sets of literature on environmental authoritarianism and the responsiveness of the Chinese government to public opinion evidently explore the same problem, namely, whether or not public opinion influences environmental governance in China. To date, there have been few qualitative studies on this topic, with most studies applying quantitative analytical approaches. Moreover, in these quantitative studies, public opinion was mainly indicated by the number of petitions, considered as an objective indicator. Consequently, there is a paucity of studies that have sought to acquire a comprehensive understanding of governmental responsiveness, considering overall public opinion as the research objective. Studies conducted by Wang and Wheeler in 2000 and 2005 constitute the main quantitative analyses on the responsiveness of China’s environmentally authoritative regime. These studies used the number of public petitions on environmental issues as an indicator of public opinion on the environment and verified governmental responsiveness by quantifying the relationship between the number of petitions and the sewage charge imposed by the government. Using objective behavioral indicators such as the number of petitions to measure public opinion on the environment usually highlights strong collective emotions of discontent relating to environmental quality. These negative emotions may also be amplified, resulting in the likely occurrence of “selectivity bias” regarding the samples and, therefore, their inability to represent universal and general conditions [25,26]. Given advances in public opinion survey technology and in the overall research environment, an assessment of environmental satisfaction based on the results of an opinion poll was conducted for this study to indicate public opinion on the environment. Subsequently, the wider and more comprehensive impacts of public opinion relating to the environment on the government’s environmental governance behaviors were investigated. The study also addresses a gap in the existing literature, namely a lack of investigation or discussion of the selectivity of responsive behaviors. Previous studies have shown that responsive governmental behaviors are selective, depending on the policy field, response objective, and channel for expressing opinions, and these findings have been widely endorsed globally [27,28,29,30,31]. Wang and Wheeler (2000) found that in the context of environmental governance in China, the government has tended to respond to the public’s environmental claims by demonstrating governance behaviors associated with atmospheric pollution and water pollution [24]. However, this phenomenon and the corresponding response mechanism have not been further investigated and discussed within subsequent studies. The current study investigated the impacts of public opinion on the environment on provincial governments’ investments in water pollution governance and waste gas pollution governance. Thus, it contributes to updating the knowledge base and advancing research on the selectivity of responsive behaviors demonstrated by the Chinese government.

The remainder of the paper is organized as follows. In Section 2, the empirical data and models applied in the study are introduced. In Section 3, empirical conclusions are posited and further explored. In addition, the results of a robustness test are presented and conclusions are offered regarding the future direction of research and development in this field. In the final section, based on the empirical conclusions of the study, some relevant policy recommendations are offered.

## 2. Materials and Methods

To determine whether the government responded to public opinion on the environment, the following fixed-effect model was developed for this study:

(1) $$I_{it}=\theta E_{it}+\beta_{1}G_{it}+\beta_{2}S_{it}+\beta_{3}S_{it}+\beta_{4}F_{it}+\beta_{5}R_{it}+\delta_{i}+\mu_{it}$$

where *i* denotes a province, *t* denotes a year, *δ_i_* is a random variable representing individual heterogeneity and a residual error for each province that does not change over time, and *μ_it_* is a residual error that changes over time against the region.

*E_it_* is the core explanatory variable—public opinion on the environment—expressed as the average value of the public’s environmental dissatisfaction within various provinces. Whereas in previous studies, the public’s opinion on the environment was mainly represented by the number of petitions related to the environment and by public opinion reports, in this study, the public’s level of environmental satisfaction was obtained from the results of a poll. On the one hand, the study entailed the use of the internationally applied empirical research technique that centers on responsiveness [28,32,33] and simultaneously addressed gaps relating to subjective indicator-related research within China. On the other hand, the public’s environmental dissatisfaction ascertained through a poll with wide coverage provided a more comprehensive description of governmental behaviors. A mutual relationship evidently exists between citizens’ opinions and governmental behaviors within a country [34,35]. Therefore, the first-order lag of the explanatory variable was regressed in the models applied in this study to separate governmental behaviors from citizens’ opinions [34,36,37].

*I_it_* denotes the explained variable, that is, the government’s pollution governance behavior represented by three indicators: the ratio of the total investment in industrial pollution governance and the total investment in fixed assets (*inv_fp*), the ratio of the total investment in industrial waste water governance and the total investment in fixed assets (*inwa_fp*), and the ratio of the total investment in industrial waste gas governance and the total investment in fixed assets (*inga_fp*). Given their availability and continuity, economic indicators such as fiscal expenditures have typically been used as indicators to represent governmental behaviors. The amplitude of their changes as well as changes in their proportions can reflect the resolution and dynamics of governmental behaviors [35].

With reference to existing theoretical and empirical studies conducted on pollution governance, other indicators that could affect local pollution governance were selected as the control variables. These were incorporated into the model to more accurately reflect the impacts of the core explanatory variable. Furthermore, the introduction of control variables can enable the avoidance of endogenous problems associated with the model and caused by missing variables.

Returning to the formula, *G_it_* denotes the level of regional economic development, represented by the regional Gross Domestic Product (GDP); *S_it_* denotes the regional industrial structure, represented by the percentage of the secondary industrial output in the regional GDP; *P_it_* denotes regional environmental pressure, represented by the size of the urban population of a region for the current year; *F_it_* denotes investments by foreign merchants in a region, represented by the percentage of foreign direct investments within the regional GDP; and *R_it_* denotes technical research and the development and innovation abilities of a region, represented by the percentage of research and development expenditure within the regional GDP. During the performance of the regression, the natural logarithms of the following variables were used to facilitate elimination of the impacts of variable units on estimated results: regional GDP and urban population size.

In the above model, the estimated coefficient of the core explanatory variable indicates whether public environmental dissatisfaction over the course of the previous year had any significant impact on the government’s pollution governance in the current year and how significant that impact was. If the estimated value is evidently negative, this means that public opinion has impacted significantly on the government’s pollution governance. If the public is dissatisfied with the local environment, this results in a tendency on the part of the government to increase pollution governance in the following year.

The core explanatory variable—public environment dissatisfaction—was derived from the results of an investigation by a research team from the Development and Research Center of the State Council on Chinese livelihood indicators, focusing on health conditions and levels of life happiness among the Chinese public [38,39]. This investigation, which was conducted through telephone interviews held from 2011 to 2015, represents one of the opinion polls with the widest coverage conducted in China. The investigation covered a total of 31 provinces, autonomous regions, and municipalities directly under the administration of the central government of China, and the respondents were urban and rural residents aged between 18 and 75 years. The total sample size was 255,278.

Table 1 presents a detailed profile of the samples. To assess environmental satisfaction among the public, the respondents were asked: “What is your evaluation of the environmental conditions at your residence (consisting of the greening rate, disposal of waste gas, liquid and residue from industrial production, and air quality)?” They were required to select their response from the following options categorized in the following order from 1 to 5: “very satisfied,” “satisfied,” “moderate,” “unsatisfied,” and “very unsatisfied.”

The explained variables and control variables were extracted from the China Yearbook and the China Yearbook of Environment and were processed for model operations as shown in Table 2.

## 3. Results

### 3.1. Governmental Responsiveness

Table 3 shows the regression relationship between public environment dissatisfaction and provincial governments’ pollution governance. The explained variable was the percentage of investments in industrial pollution governance in relation to investments in fixed assets, and the explanatory variable was the first-order lag of the average level of local environmental dissatisfaction. A stepwise regression method was adopted and the regression results of each step were showed.

The estimated results for models (1)–(4) revealed that the public’s dissatisfaction with the environment had a positive impact on investments in industrial pollution governance at a significance level of *p* < 0.05. In other words, if the public was not satisfied with the environment during a particular year, then the local government would increase its investment in pollution governance during the following year. More specifically, when the public’s evaluation of the environment declined from “very unsatisfied” (*E* = 5) to “very satisfied” (*E* = 1), the percentage of local investments in industrial pollution governance in relation to total investments in fixed assets decreased by 0.776%. This figure was 5.5 times above the national average (0.14%) and 5.8 times above the national median (0.13%) in 2015. Data extracted for the period 2011–2015 showed change equated to 6.7 standard deviations from the explained variable. Several key conclusions emerged from these results. First, in terms of local governments’ investments in industrial pollution governance, public opinion on the environment had a significant impact on pollution governance. The situation in 2015 can be considered as an example. Assuming other factors remained unchanged, if local governments wanted to induce a change in the public’s environmental satisfaction from “very satisfied” (*E* = 1) to “very unsatisfied” (*E* = 5), then over half of local governments would increase the percentage of their industrial pollution governance investments in fixed assets by more than 5.8 times, and over 90% of local governments would increase this percentage by more than 3.6 times.

The regressions performed for Models (1)–(4) indicated that the natural logarithms of the regional GDP and the urban population size yielded significant results at a significance level of *p* < 0.1. Models (2)–(4) generated positively significant results for regional GDP, which represents local economic development, indicating that the percentage of investments in environmental governance was high in economically developed regions. The estimation result for model (3) was negatively significant for urban population size, which represented objective environmental pressure within a region. This indicated that environmental pressure was greater in regions with larger populations, which required higher percentages of investments in environmental pollution governance.

### 3.2. Selectivity of Responsive Behavior

Previous studies have indicated the selectivity of responsive governmental behaviors [35,40]. Considering the urgency and sustained attention that atmospheric pollution governance has received from the public, the responses of local governments to atmospheric and water pollution were analyzed and compared in this study. A stepwise method was used to estimate governance responses to public opinion on atmospheric and water pollution. The results are shown in Table 4.

The explained variable for models (1)–(3) was the percentage of investments in industrial waste gas governance in fixed investment. The core explanatory variable was also the first-order lag of the average value of the public’s environmental dissatisfaction. The regression results showed that the public’s environmental dissatisfaction was positively significant in relation to the provincial governments’ investments in waste gas governance at a significance level of *p* < 0.05. In other words, a higher level of environmental dissatisfaction within the public corresponded to increased investments by local governments in atmospheric pollution governance. The regression results for model (3) showed that with a change in the level of the public’s environmental dissatisfaction from “very satisfied” (*E* = 1) to “very unsatisfied” (*E* = 5) the percentage of local governments’ investments in industrial waste gas pollution governance increased by 0.78%. Once again taking 2015 as an example, the regression results indicated that over half of the provincial governments had to increase their investments in waste gas governance by more than ninefold, and nearly 90% of the provincial governments had to increase their total investments in waste gas pollution governance by more than fivefold.

The explained variable for models (4)–(6) was the percentage of governmental investments in industrial waste water governance in relation to fixed investment. The core explanatory variable was also the first-order lag of the average value of the public’s environmental dissatisfaction. None of the regression results were highly significant for the core explanatory variable, namely environment dissatisfaction, indicating that governments seldom invested in water pollution governance in response to public opinion.

The governments’ actual investments in atmospheric pollution and water pollution governance revealed their selective responses to public opinion on the environment. These responsive behaviors can be attributed to the high visibility and easy availability of information on atmospheric pollution that have prompted a high level of public concern and focused attention on air quality. Wheeler and Dasgupta (2016) pointed out that public complaints were associated with visibility rather than the actual hazards of pollution [41]. People can physically witness atmospheric pollution as well as consult mobile apps to acquire knowledge of accurate pollution indicators. Consequently, the public is familiar with atmospheric pollution, which evokes strong emotions of discontent. According to the CEY, 40% of petition letters relating to long-term petition work conducted in various areas focused on atmospheric pollution. This figure was more than 10% higher than that of the second most pressing environmental concern, namely water pollution.

### 3.3. Robustness Check

To ensure the robustness of the evaluation results, a method for changing the core explanatory variable indicators was applied for verification purposes. In order to replace the core explanatory variables, the mean of the 20% dissatisfaction of the environmental dissatisfaction of the provinces (*L.a_20*) and the mean of the reciprocal of public environmental satisfaction in each province (*L.v_se*) were taken, and the first-order lag was used. The estimation results shown in Table 5 were obtained through the adoption of first-order lag and the application of stepwise regression.

The robustness check results showed that the estimation coefficient used in the empirical analysis was reasonably robust. In models (1)–(3), the core explanatory variable was the first-order lag of the mean value of dissatisfaction for 20% of the provinces with the original, unchanged explained variable and the highest degree of public dissatisfaction with the environment. The regression results indicated that the explanatory variable always remained positively significant at a significance level of *p* < 0.05. The estimated coefficient was slightly lower than the regression results for the satisfaction value, but the significance and estimated values of the models were the same as those in the original model. In models (4)–(6), the core explanatory variable was the first-order lag of the mean value of the reciprocal for the public’s environmental dissatisfaction in all provinces, with the original, unchanged explained variable and the highest degree of public dissatisfaction with the environment. The regression results indicated that the core explanatory variable always remained negatively significant at a significance level of *p* < 0.05. In other words, when the public was more unsatisfied with the environment, local governments increased their investments in environmental governance. All six models have been validated by Wald and Hausman tests. The results showed that the fixed-effect model was applicable to the above regression results and that it presented a superior option compared with the mixed least square method and the random effect model.

The same alternative indicators for testing were adopted as those applied in the empirical analysis on the selectivity of responsive governmental behaviors. Table 6 and Table 7 present the results of these tests. Thus, the robustness of the analysis results on the selectivity of the provincial governments’ responsive behaviors was verified.

Furthermore, we need to offer alternative explanations and potential concerns of endogeneity, such as the baseline pollution rates. Table 8 shows that the influence of the two baseline pollution rates is an important factor and adding these variables does not affect the significance and coefficient of the main explanatory variables, which indicates that the empirical work is robust, since considering the pollute gas (represented by dust emissions (We have used different indexes, such as sulfur dioxide emissions, nitrogen oxide emissions, and get the similar results. Due to limited space, we cannot report all the empirical results, but you can ask us for more information.), *pollu_ga*) and pollute water (*pollu_wa*) annual data by region from the Nation Bureau of Statistics of China.

## 4. Discussion

The Chinese government will not respond to the national surveys on the public’s environmental satisfaction administered during the period 2011–2015. Only when the government wants to give feedback, the government modifies its behavior, there are following two reasons. First, the Chinese ideology requires that the Chinese government respond equally to all types of public opinion. However, it is difficult for the Chinese government to make decisions and respond comprehensively to all public opinions, due to the lack of competitive-selection mechanism. Therefore, the Chinese government tends to put more efforts into the environmental field where it could more easily achieve short-term benefits. Second, as air pollution is less likely to be protected and is more equitable, the mass is more sensitive to air pollution. Faced with more and more unsatisfied appeals and complaints, the government feels greater pressure. As a result, the government will spend more time and funds on air pollutant protection.

Through the above analysis about the relationship of the Chinese government’s responsive behavior and public opinion, future study needs to take the “decentralized” authoritarian political structure [42] into consideration and increase discussion on the game theory among central government, local government, and public opinion.

It includes the central government influence, the game theory between the central and local government and its impact on the feedback given by the central government to the public opinion. Furthermore, when taking China’s vast territory, diversity and different economic developments into consideration, the central government will give different responses to different areas; the local government also will choose different strategies to balance the pressure from the central government and the public opinion.

In addition, due to the limitations of the questionnaire, the research also has some space to improve, which mainly includes two points. One is that the research cannot directly distinguish public opinions on water pollution and air pollution and, having to evaluate them as a uniform variable of environmental satisfaction, it may make some inaccurate judgment about the governance feedback to the different public environment product preferences. The other is that the research cannot clearly distinguish the incentive the local government is given by the central government, or from the public opinion directly. It needs more detailed studies in the future. The findings of this study could be extended and applied to other countries, especially for those countries that have separate and responsive behaviors on both air pollution and water pollution. This discovery of separate treatments on both air and water pollution assists in the investigation into more detailed discussion on public goods in different types of governance in the further regime of environmental governance research. This study also helps to explain the interaction between the authoritative country’s behavior and the public opinion, which is not limited to the environmental field, but also could apply to all of the public goods areas, such as public education and health care.

## 5. Conclusions

The findings of the empirical study presented in this paper reveal that in the field of environmental governance, the Chinese government’s responsive behavior relating to public opinion on the environment demonstrated the following three characteristics. First, the Chinese provincial level governments do respond to public opinion on the environment. If the public is not satisfied with the environment in a given year, then the concerned local government will increase its investment in pollution governance during the following year. Conversely, if public satisfaction with environmental quality increases in a given year, then the concerned local government will reduce its investment in pollution governance during the following year. Second, Chinese provincial level governments’ responsive behavior is selective in relation to public opinion on the environment. This characteristic of selectivity associated with responsive governmental behavior is also reflected in China’s environmental governance. The government does not respond equally to all types of public opinion and will change its response based on the objective of governance.

The last characteristic entails the tendency of the Chinese government to respond to public opinion on the environment through atmospheric pollution governance behaviors rather than water pollution governance behaviors. The findings of this empirical study revealed that when the public was less satisfied with the environment, the concerned local government subsequently increased its investment in atmospheric pollution governance. However, investments in water pollution governance evidently did not change. This is because atmospheric pollution is the most visible type of pollution. Moreover, because information-gathering channels are most accessible for this form of pollution, it is more easily recognizable by the public compared with other forms of pollution. Therefore, atmospheric pollution tends to evoke emotional reactions and discontent within the public, whereas improvements in air quality tend to evoke calmer reactions associated with greater public satisfaction with environmental quality within the shortest time.

The above three conclusions are derived from empirical research and augment the literature on the responsiveness of China’s environmental governance. Through its quantitative analysis of existing research results, the present study has advanced knowledge in this field. Moreover, its findings present updated and confirmed research outcomes on the selectivity of the Chinese government’s responsive behaviors within the field of environmental governance.

Based on the above three conclusions, the following recommendations can be made for improving both policy and governance to better utilize the responsive effect of the government and further promote China’s environmental governance levels. First, there is a need to improve the transparency of environmental information and guarantee the public’s right to know. Accomplishment of these goals would result in an improved information base that is accessible to members of the public who seek to express their opinions. The provision of incomplete information results in a lower level of public awareness regarding pollution. Consequently, it is important to recognize and guarantee the public’s right to know in relation to environmental information. Accordingly, the provision of public environmental information products should be enhanced to facilitate a more comprehensive understanding of environmental conditions among members of the public. This would allow citizens to formulate demands for improvement that are aligned with actual environmental conditions. These informed demands, in turn, would serve to guide the government in effecting a more rational distribution of funds for pollution governance and adopting more scientific environmental governance measures.

In addition, there is a need to improve channels that facilitate the public’s participation in environmental governance. This would help to ensure that members of the public can fully and comprehensively express their environmental opinions. At present, the system for including public participation within China’s environmental governance process comprises single, systematically unsound channels with narrow coverage. To rectify this situation, further improvements and the advancement of the environmental governance system incorporating public participation, with environmental petitioning as its core, is required. Moreover, a system for promoting public participation in environmental impact evaluations and in hearings on major environmental events should be the main drivers. Further, diverse channels such as leaders’ mail boxes and hotlines, government consultancy platforms, polls, and reports on public opinion should be promoted and connected to various sectors related to the environmental policy development process. Efforts are also required to guide the public’s rational participation in policy decision making, implementation, and monitoring following implementation.

To better reflect the public’s satisfaction regarding environmental governance, a final recommendation is to further improve the performance evaluation system for assessing officials in the field of environmental governance. An examination of existing environmental governance practices reveals that the assessment system of the governors is still the major factor influencing the implementation of environmental policies. Reforming the system’s design and incorporating factors associated with public satisfaction with the environment would be the most effective measure for improving governmental responsiveness. Although it is relatively difficult to directly incorporate public satisfaction within the assessment system for officials over the short term, performance relating to environmental governance has become part of the system for assessing officials. Thus, indirect indicators of public environmental satisfaction will be gradually reflected within this system. For example, it is feasible and logical to incorporate the number of environmental petition cases along with performance in environmental governance. This measure would enable further progress of the evaluation system for officials toward more “people-oriented” governance and development. This should be done with a focus on “green development,” as advocated by the Communist Party of China, and would help to alleviate conflict caused by existing discrepancies between the system and ideology that are expressed in the behaviors of officials.

## Figures and Tables

**Table 1:** Sample structure of telephone interview.

Item	Option	Sample Size	Sample Percentage (%)
Region	Cities and towns	159,022	62.29
	Rural area	96,256	37.71
Gender	Male	131,216	51.40
	Female	124,062	48.60
Education background	Primary school and below	34,995	13.71
	Junior high school	66,311	25.98
	High school	67,819	26.57
	Junior college	44,033	17.25
	Undergraduate	38,792	15.20
	Postgraduate and above	3327	1.30
Age	18–24	24,530	9.61
	25–34	57,516	22.53
	35–44	55,643	21.80
	45–54	46,275	18.13
	55–64	32,291	12.65
	65–75	20,082	7.87
Year	2011	51,100	20.02
	2012	51,100	20.02
	2013	51,067	20.00
	2014	50,994	19.98
	2015	51,017	19.98

**Table 2:** Data Collecting and Process.

Variable	Indicator	Source
Public environmental opinion/*E_it_*	The first-order lag of the average value of public environmental dissatisfaction in every province/*L.dis_envir*	Chinese livelihood indicator research team
-	The ratio between total investment in industrial pollution governance and total investment in fixed assets/*inv_fp*	China Yearbook and China Yearbook of Environment
Government pollution governance behavior/*I_it_*	The ratio between the investment in industrial waste water governance and the total investment in fixed assets/*inwa_fp*	China Yearbook and China Yearbook of Environment
-	The ratio between the investment in industrial waste gas governance and investment in fixed assets/*inga_fp*	China Yearbook and China Yearbook of Environment
Regional economic development level/*G_it_*	The natural logarithm processing of regional Gross Domestic Product (GDP)/*ln_gdp*	China Yearbook
Regional industrial structure/*S_it_*	The percentage of second industrial output in the regional GDP/*ind_p*	China Yearbook
Regional environmental pressure/*P_it_*	The natural logarithm processing of the urban population numbers of a region for the current year/*ln_pop_u*	China Yearbook
Regional investment from foreign merchants/*F_it_*	The percentage of foreign direct investment in the regional GDP/*fdi_pp*	China Yearbook
Technical research and the development and innovation ability of a region/*R_i_*	The percentage of research and development expenditures in the regional GDP/*rd*	China Yearbook

**Table 3:** Estimation results of the total investment response in industrial pollution governance vis-à-vis public environmental dissatisfaction.

Explained Variable	Industrial Pollution Governance	(1)	(2)	(3)	(4)
Explanatory variable	*L.dis_envir*	0.155 **	0.218 **	0.205 **	0.193 **
		(0.063)	(0.082)	(0.079)	(0.087)
Control variable	*ln_gdp*		0.157 *	0.648 ***	0.576 ***
			(0.087)	(0.141)	(0.173)
	*ind_p*			0.140	0.608
				(0.464)	(0.495)
	*ln_pop_u*			−1.322 ***	−0.929
				(0.359)	(0.558)
	*fdi_pp*				−0.014
					(0.031)
	*rd*				0.011
					(0.021)
Constant term	Constant	−0.244	−1.560 *	4.717 **	2.144
		(0.172)	(0.800)	(2.169)	(3.267)
Sample size	Observations	124	124	123	111
Goodness of fit	*R*-squared	0.062	0.092	0.187	0.175
Number of provinces	No. of prov.	31	31	31	30

Note: the standard error of each estimated value is provided in brackets; *** represents *p* < 0.01, ** represent *p* < 0.05 and * represents *p* < 0.1.

**Table 4:** Results of the response in waste water and waste gas governance investment vis-à-vis public environmental dissatisfaction.

Variables	Waste Gas			Waste Water		
		(1)	(2)	(3)	(4)	(5)	(6)
*L.dis_envir*	0.111 **	0.212 ***	0.194 ***	0.018	−0.021	−0.027
	(0.040)	(0.062)	(0.065)	(0.013)	(0.017)	(0.020)
*ln_gdp*		0.577 ***	0.635 ***		−0.026	−0.106 *
		(0.106)	(0.145)		(0.045)	(0.062)
*ind_p*		0.120	0.265		−0.017	0.165
		(0.319)	(0.399)		(0.099)	(0.152)
*ln_pop_u*		−0.800 ***	−0.853 *		−0.184 **	0.099
		(0.220)	(0.449)		(0.079)	(0.179)
*fdi_pp*			0.011			−0.015
			(0.026)			(0.010)
*rd*			0.015			−0.003
			(0.023)			(0.008)
Constant	−0.181	1.260	1.179	−0.020	1.663 ***	0.100
	(0.110)	(1.327)	(2.687)	(0.035)	(0.517)	(1.061)
Observations	124	123	111	124	123	111
*R*-squared	0.043	0.216	0.229	0.012	0.168	0.207
No. of prov.	31	31	30	31	31	30

Note: the standard error of each estimated value is provided in brackets; *** represents *p* < 0.01, ** represent *p* < 0.05 and * represents *p* < 0.1.

**Table 5:** Robustness test on the industrial pollution governance investment response vis-à-vis public environment dissatisfaction.

Industrial Pollution Governance	Waste Gas			Waste Water		
	(1)	(2)	(3)	(4)	(5)	(6)
*L.a_20*	0.150 ***	0.163 **	0.150 **			
	(0.052)	(0.062)	(0.068)			
*L.v_se*				−0.897 **	−0.870 *	−0.810
				(0.406)	(0.431)	(0.484)
*ln_gdp*		0.595 ***	0.543 ***	0.132	0.649 ***	0.585 ***
		(0.130)	(0.167)	(0.092)	(0.151)	(0.184)
*ind_p*		0.290	0.757		0.185	0.657
		(0.455)	(0.491)		(0.486)	(0.496)
*ln_pop_u*		−1.255 ***	−0.930		−1.360 ***	−0.984
		(0.357)	(0.552)		(0.378)	(0.585)
*fdi_pp*			−0.018			−0.015
			(0.032)			(0.031)
*rd*			0.008			0.014
			(0.020)			(0.021)
Constant	−0.474 **	4.388 *	2.209	−0.385	5.919 **	3.354
	(0.225)	(2.225)	(3.268)	(0.562)	(2.158)	(3.304)
Observations	124	123	111	124	123	111
*R*-squared	0.072	0.183	0.167	0.073	0.177	0.165
No. of prov.	31	31	30	31	31	30

Note: the standard error of each estimated value is provided in brackets; *** represents *p* < 0.01, ** represent *p* < 0.05 and * represents *p* < 0.1.

**Table 6:** Robustness check on the waste gas governance response vis-à-vis public environmental dissatisfaction.

Variables	Waste Gas					
		(1)	(2)	(3)	(4)	(5)	(6)
*L.a_20*	0.128 ***	0.177 ***	0.161 ***			
	(0.041)	(0.049)	(0.054)			
*L.v_se*				−0.521 **	−0.973 ***	−0.890 **
				(0.217)	(0.342)	(0.371)
*ln_gdp*		0.521 ***	0.601 ***		0.582 ***	0.647 ***
		(0.093)	(0.138)		(0.117)	(0.153)
*ind_p*		0.265	0.403		0.142	0.290
		(0.296)	(0.383)		(0.330)	(0.391)
*ln_pop_u*		−0.724 ***	−0.847 *		−0.837 ***	−0.905 *
		(0.206)	(0.423)		(0.236)	(0.467)
*fdi_pp*			0.006			0.010
			(0.026)			(0.026)
*rd*			0.011			0.018
			(0.024)			(0.022)
Constant	−0.436 **	0.841	1.163	0.355 ***	2.508 *	2.397
	(0.179)	(1.273)	(2.503)	(0.098)	(1.292)	(2.682)
Observations	124	123	111	124	123	111
*R*-squared	0.072	0.220	0.228	0.043	0.214	0.228
No. of prov.	31	31	30	31	31	30

Note: the standard error of each estimated value is provided in brackets; *** represents *p* < 0.01, ** represent *p* < 0.05 and * represents *p* < 0.1.

**Table 7:** Robustness check on the waste water governance response vis-à-vis public environmental dissatisfaction.

Variables	Waste Water					
		(1)	(2)	(3)	(4)	(5)	(6)
*L.a_20*	0.009	−0.016	−0.022			
	(0.016)	(0.020)	(0.022)			
*L.v_se*				−0.074	0.096	0.121
				(0.065)	(0.084)	(0.099)
*ln_gdp*		−0.020	−0.102 *		−0.026	−0.108 *
		(0.042)	(0.059)		(0.045)	(0.063)
*ind_p*		−0.033	0.146		−0.019	0.161
		(0.101)	(0.146)		(0.096)	(0.149)
*ln_pop_u*		−0.191 **	0.098		−0.181 **	0.106
		(0.076)	(0.171)		(0.080)	(0.183)
*fdi_pp*			−0.014			−0.015
			(0.010)			(0.010)
*rd*			−0.003			−0.003
			(0.008)			(0.008)
Constant	−0.009	1.690 ***	0.101	0.063 **	1.539 ***	−0.069
	(0.071)	(0.510)	(1.007)	(0.030)	(0.509)	(1.105)
Observations	124	123	111	124	123	111
*R*-squared	0.004	0.167	0.207	0.009	0.168	0.206
No. of prov.	31	31	30	31	31	30

Note: the standard error of each estimated value is provided in brackets; *** represents *p* < 0.01, ** represent *p* < 0.05 and * represents *p* < 0.1.

**Table 8:** Robustness check on the potential concerns of endogeneity.

Industrial Pollution Governance	Inv_fp		Inga_fp		Inwa_fp	
	(1)	(2)	(3)	(4)	(5)	(6)
*L.a_20*	0.149 **		0.158 ***		−0.021	
	(0.064)		(0.046)		(0.025)	
*L.v_se*		−0.865 *		−0.963 **		0.132
		(0.488)		(0.361)		(0.097)
*ln_gdp*	0.611 ***	0.633 ***	0.687 ***	0.710 ***	−0.106 *	−0.109 *
	(0.157)	(0.168)	(0.119)	(0.129)	(0.061)	(0.063)
*ind_p*	0.818	0.818	0.511	0.499	0.124	0.127
	(0.542)	(0.534)	(0.386)	(0.391)	(0.156)	(0.150)
*ln_pop_u*	−1.034 **	−1.107 **	−0.989 ***	−1.065 ***	0.112	0.122
	(0.489)	(0.507)	(0.338)	(0.365)	(0.167)	(0.177)
*fdi_pp*	−0.015	−0.008	0.011	0.020	−0.015	−0.016
	(0.032)	(0.032)	(0.027)	(0.027)	(0.010)	(0.010)
*rd*	−0.005	0.003	−0.005	0.003	−0.002	−0.003
	(0.017)	(0.021)	(0.018)	(0.019)	(0.007)	(0.006)
*pollu_wa*	−0.011 **	−0.009	−0.014 ***	−0.011 **	0.001	0.001
	(0.005)	(0.006)	(0.004)	(0.005)	(0.002)	(0.002)
*pollu_ga*	0.001	0.002	0.001 **	0.002 **	−0.000	−0.000
	(0.001)	(0.001)	(0.001)	(0.001)	(0.000)	(0.000)
Constant	2.683	4.001	1.841	3.244	0.021	−0.166
	(2.918)	(2.920)	(2.055)	(2.186)	(1.010)	(1.082)
Observations	111	111	111	111	111	111
*R*-squared	0.189	0.195	0.279	0.296	0.212	0.217
No. of prov.	30	30	30	30	30	30

Note: the standard error of each estimated value is provided in brackets; *** represents *p* < 0.01, ** represent *p* < 0.05 and * represents *p* < 0.1.
